# Sequential Immune Thrombocytopenia (ITP) and Thrombotic Thrombocytopenic Purpura (TTP) in an Elderly Male Patient with Primary Sjogren's Syndrome: When in Doubt, Use the PLASMIC Score

**DOI:** 10.1155/2021/6869342

**Published:** 2021-11-30

**Authors:** Devon D. Miller, Joseph A. Krenzer, Vaishalee P. Kenkre, William Nicholas Rose

**Affiliations:** University of Wisconsin Hospital and Clinics, 600 Highland Ave, Madison, WI 53792, USA

## Abstract

**Introduction:**

Thrombotic thrombocytopenic purpura (TTP) is a rare, life-threatening thrombotic microangiopathy due to an acquired autoantibody to ADAMTS13 that requires a boutique treatment, urgent plasma exchange. Thus, TTP is often termed a “cannot miss” diagnosis.

**Case:**

We report a patient with TTP who had a history of immune thrombocytopenia (ITP), had atypical demographics for TTP, and had also met criteria for primary Sjogren's syndrome. This exceedingly rare combination presented a temptation to dismiss TTP as a diagnosis. *Discussion*. Our case further demonstrates the practical utility of using the PLASMIC score as a tool that can help identify patients with TTP even when the patient has statistically rare characteristics.

## 1. Introduction

Immune thrombocytopenia (ITP) and thrombotic thrombocytopenic purpura (TTP) are two separate diseases that are characterized by low platelets.

Immune thrombocytopenia (ITP) is an acquired hematologic disorder caused by immune-mediated destruction of platelets that can be classified as idiopathic or secondary to another disease process. ITP is a clinical diagnosis that rests on the presence of isolated thrombocytopenia while excluding other causes of thrombocytopenia and identifying conditions that may be causing secondary ITP [1].

Thrombotic thrombocytopenic purpura (TTP) is a rare thrombotic microangiopathy caused by an acquired autoantibody that leads to decreased activity of the von Willebrand factor-cleaving protease ADAMTS13 that results in hemolytic anemia and severe thrombocytopenia [2]. While the treatment courses for both ITP and TTP may include corticosteroids and rituximab, a key treatment difference is urgent therapeutic plasma exchange (TPE) for all patients with TTP, whereas TPE is not effective for ITP.

In this article, we report a case of sequential ITP and TTP in a patient with primary Sjogren's syndrome who was diagnosed in part due to the utility of the PLASMIC scoring system.

## 2. Case Presentation

**Figure 1:**
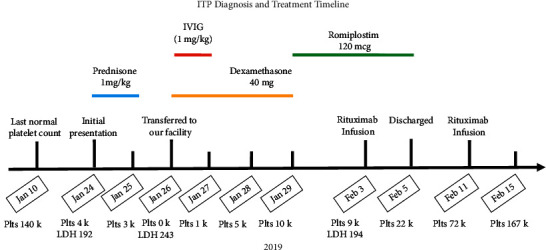
ITP diagnosis and treatment timeline. Major dates in the patient's initial ITP presentation and treatment course occurring in 2019 are listed along the *x*-axis with accompanying hash marks and events above. Daily platelet counts (/*µ*L) and LDH (U/L) where available are listed below respective dates. The above timeline includes major therapies with which the patient was treated, spanning across dates received including prednisone 1 mg/kg (blue bar), IVIG (red bar), dexamethasone 40 mg (yellow bar), and romiplostim 120 mcg (green bar). Overlapping bars imply multiple treatments given on same days.

A 72-year-old Caucasian man was diagnosed in 2019 with ITP after he presented to his local emergency department (ED) with bruising on his left arm, transient hematuria, melena, persistent epistaxis, and a platelet count of 4,000/*µ*L. A complete blood count showed that his hemoglobin level was 14.0 g/dL and also confirmed the severe thrombocytopenia. Hemolysis labs showed LDH 192 U/L, total bilirubin 0.4 mg/dL, haptoglobin 52 mg/dL, and reticulocyte count 1.6%. His presentation was felt to be drug-induced ITP from apixaban that was started several months earlier. He received 1 mg/kg prednisone and was transfused 1 unit of platelets before being admitted. Despite steroids and 2 additional platelet transfusions over the next two days, his platelets remained 4,000/*µ*L, prompting transfer to our facility for IVIG consideration (Figure 1).

On arrival, his platelet count was 0k/*µ*L. His peripheral smear showed thrombocytopenia with giant platelets, some rouleaux formation, occasional large granular lymphocytes with otherwise normal red and white cell morphology, and no fragmented cells or schistocytes. His workup was significant for elevated rheumatoid factor to 71 IU/ml, ANA positive at >1:640 with a speckled pattern, ESR of 53 mm/hr, negative lupus anticoagulant, negative cardiolipin antibody, negative beta-2 glycoprotein, negative hepatitis C, negative hepatitis B, and negative apixaban-dependent platelet antibody. Bone marrow biopsy showed normocellular age with normal megakaryocytes.

The patient was treated with dexamethasone 40 mg × 4 days and IVIG 1 g/kg × 2. His platelet count increased to 12,000/*µ*L. Following this therapy, his platelet count dropped to 5,000/*µ*L. He received romiplostim 120 (1 mcg/kg) mcg daily. After 6 days of romiplostim, his platelet count was 9,000/*µ*L, so he received rituximab (375 mg/m2; 850 mg infusion) as well as mycophenolate (1,000 mg twice daily). At discharge, his platelet count was 22,000/*µ*L, and he was continued on romiplostim 2 mcg/kg, mycophenolate, and rituximab. One week later, his platelet count was 72,000/*µ*L, and he received one additional rituximab infusion before being followed with observation. Over the next 13 months, his platelet count was between 74,000/*µ*L and 157,000/*µ*L.

In September 2020, nearly 19 months after initially presenting with ITP, our patient presented to his local ED with hematuria and epistaxis again. His platelet count was 9,000/*µ*L, hemoglobin was 12 g/dL, and total bilirubin was 5.3 mg/dL. A relapse of his ITP was diagnosed. He was initially treated with dexamethasone 8 mg and transferred to our facility for possible rituximab infusion. On arrival, his platelet count was 2,000/*µ*L, hemoglobin was 11.1 g/dL (down from a baseline of 15), and schistocytes were seen on peripheral smear. He had a new acute kidney injury with a creatinine of 2.41 mg/dL (up from a baseline of 1 mg/dL) and an LDH of 2,399 U/L. A haptoglobin level was undetectable, and reticulocyte count was 2.1%.

The new hemolytic anemia as well as the severe thrombocytopenia raised the possibility of TTP. The patient had several characteristics that were statistically uncommon for TTP patients. Namely, he was male, in his seventies, and Caucasian. Furthermore, he had an existing diagnosis of ITP as well as symptoms that were nearly identical to his previous presentation with ITP. However, ITP alone would not explain his hemolytic anemia. Thus, the PLASMIC score was used, and his score was high. This meant that the probability of TTP was high based on this scoring system.

**Figure 2:**
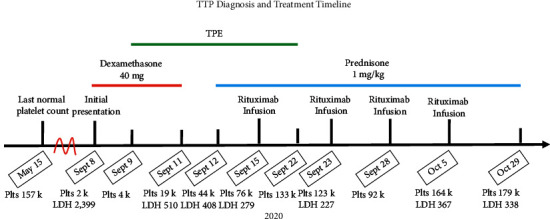
TTP diagnosis and treatment timeline. Major dates in patient's TTP presentation and treatment course occurring in 2020 are listed along the *x*-axis with accompanying hash marks and events above. The red scale break on the *x*-axis signifies large relative gap in time between dates. Daily platelet counts (/*µ*L) and LDH (U/L) where available are listed below respective dates. The above timeline includes major therapies with which the patient was treated, spanning across dates received including dexamethasone 40 mg (red bar), prednisone 1 mg/kg (blue bar), and plasma exchange (TPE, green bar). Overlapping bars imply multiple treatments given on same days.

Given the high likelihood for TTP, he received urgent plasmapheresis (Figure 2). He also received prednisone (1 mg/kg; 110 mg). The diagnosis of TTP was confirmed during his first plasmapheresis by a severely low ADAMTS13 activity of 9% as well as the presence of an inhibitor.

Given his positive rheumatoid factor, positive ANA, and multiple autoimmune conditions, rheumatology was also consulted. He had a positive SSA and positive Schirmer's test of the left eye. Thus, he met ACR/EULAR classification criteria for primary Sjogren's syndrome. Given his atypical demographics for Sjogren's and lack of classic sicca symptoms, a labial salivary gland biopsy was performed but was not consistent with Sjogren's syndrome. Rheumatology suggested he may have Sjogren's secondary to SLE given  the positive ANA, low C4, and association between SLE and TTP. However, his dsDNA, RNP, Smith, and APS were all negative.

His hospital course was complicated by MSSA bacteremia.This was presumably due to his apheresis/dialysis central venous catheter, so it was removed. Both TTE and TEE showed no evidence of vegetations. He was started on cefepime before narrowing to cefazolin with an outpatient plan of daptomycin for 4 weeks of total antibiotic therapy.

He received daily TPE x14. He also received rituximab (375 mg/m2; 800 mg infusion) once a week x4. Dose 1 of this series was after TPE #7, dose 2 was after TPE #14, and doses 3 and 4 were over the following 2 weeks. Eventually, his platelets plateaued to around 120,000/uL. This was close to his baseline of around 130,000–150,000/uL during the previous year after he had been treated for ITP. He was discharged and did well at home. A month after discharge, his platelets increased to 179,000/uL without any significant drops.

## 3. Discussion

ITP is a diagnosis of exclusion made in patients with isolated thrombocytopenia due to immune-mediated destruction of platelets. The diagnosis of ITP includes careful evaluation of the patient's history and physical examination along with laboratory testing including review of the peripheral smear to rule out other potential causes of thrombocytopenia. The pathogenesis of ITP is incompletely understood. Antibody-mediated destruction is generally considered the most common cause, as 60–70% of patients have platelet-specific immunoglobulin G autoantibodies, most commonly to platelet glycoprotein IIb/IIIa complex [1]. Other reported mechanisms include T-cell-mediated destruction of platelets [3] and impaired megakaryopoiesis [4]. Despite these frequent antibody associations, current antibody tests have been consistently demonstrated to be poorly sensitive and poorly specific for ITP in nearly all studies that have been published. Thus, ITP is a clinical diagnosis since no widely validated antibody test exists as of this writing.

In 80% of ITP cases, the cause is idiopathic. The other 20% of cases are secondary to conditions such as autoimmune syndromes, immunodeficiency syndromes, infections, lymphoid malignancies, and medications [5]. Treatment for newly diagnosed primary ITP is typically reserved for patients with clinically significant bleeding and severely low platelets (<30,000/microL), as the overall risk for bleeding in ITP is low. The primary first-line treatment of ITP is corticosteroids. Increased or normalized platelet counts are usually seen within two weeks, and this response to therapy is sometimes used in practice as a confirmatory test of sorts.

TTP is also a disorder that is characterized by thrombocytopenia, but TTP includes microangiopathic hemolytic anemia (MAHA) in addition to severe thrombocytopenia. While the pentad of fever, neurological and renal abnormalities, MAHA, and thrombocytopenia is classically associated with TTP, the full pentad is only seen in 5% of cases and should not be relied upon as a sensitive set of criteria for diagnosis [2, 6]. In addition, while schistocytes or red cell fragments are often seen in the disease, their presence is neither sensitive nor specific for TTP [7, 8]. In our experience, it is often a pitfall to rely on the presence or absence of overt end-organ damage or schistocytes.

TTP is characterized by widespread small-vessel platelets and von Willebrand factor-rich thrombi that are the result of a reduction in the activity of the enzyme ADAMTS13, a disintegrin and metalloproteinase involved in cleavage of large vWF multimers [9]. Severe ADAMTS13 deficiency (<10%) is very often seen in TTP [2] and is caused by an autoantibody (often reported by the lab as an inhibitor) that is commonly detected using a mixing study [10].

The primary treatment of TTP is urgent TPE followed by TPE daily until the platelet count normalizes. Before the discovery of TPE as the treatment, TTP was fatal in 90% of patients [9]. Today, with treatment, about 80–90% of patients survive [2].

Recently, the diagnostic accuracy of the PLASMIC score was validated by a systematic review involving nearly 1,000 patients [11]. The PLASMIC score ranges from 0–7 and gives one point each for the following: the absence of active cancer, the absence of solid organ and stem cell transplant, and five categories of laboratory results. A PLASMIC score of 5 or greater has a sensitivity of 99 percent and specificity of 57 percent for ADAMTS13 deficiency. Given the potentially life-threatening results of withholding therapy as well as the relative safety of TPE, treatment with urgent TPE is recommended for patients with a score of 5 or higher while awaiting results of ADAMTS13 testing. We hasten to add that the turnaround time of ADAMTS13 testing has dramatically improved recently in many settings due to the wider availability of in-hospital test kits. Thus, guidelines that recommend TPE for patients with a low pretest probability of TTP usually assume that a turnaround time of at least a few days is required for a reference lab to perform the test. This is essentially rendered moot when an in-hospital test can produce a result within 45 minutes to a few hours. In other words, a diagnosis of “can't rule out TTP” in patient with a low PLASMIC score can very often be ruled out by a rapid in-hospital test (and infusing plasma while waiting, if clinically indicated). Awareness of this innovation should be raised, especially when writing guidelines.

The exact diagnosis (or diagnoses) of our patient is open to debate and speculation. Sequential or concomitant ITP and TTP have been previously described in the literature [12, 13] and have been associated with pregnancy [14, 15], HIV [16–18], and essential thrombocythemia [19]. The 2001 review by Baron et al. identified 11 cases of sequential or concurrent ITP and TTP without HIV and demonstrated a female predominance (9/11, 81%) and an association with pregnancy and autoimmune disorders including systemic lupus erythematosus (SLE), rheumatoid arthritis (RA), hypothyroidism, and Sjogren's syndrome secondary to RA [13].

Our patient met ACR diagnostic criteria for primary Sjogren's syndrome (pSS) during his most recent hospitalization. While secondary ITP in patients with pSS may be relatively common [20], secondary TTP in a male patient with pSS is exceedingly rare. The recent review by Carvalho et al. identified 18 patients with SS and TTP [21]. pSS was observed in 16/18 (88.9%) with a similar female predominance (15/18, 83%) to patients with both ITP and TTP. In most cases they reviewed, SS preceded TTP. This may fit the timeline of our patient, as he was presumed to have an autoimmune condition at his first presentation that was likely to be pSS and later diagnosed during the subsequent hospitalization.

Due to the increased co-occurrence of both ITP and TTP in patients with autoimmune conditions, HIV, or pregnancy, it has been hypothesized that there may be some shared pathophysiologic factors such as redundancy of the immune system or a mixed immune thrombocytopenia syndrome that connects the two conditions [13, 22]. A recent article described 19 novel autoantibodies in Sjogren's syndrome [23] that may further explain the connection between ITP, TTP, and secondary autoimmune conditions.

To our knowledge, this is the first case of sequential ITP and TTP associated with pSS, and it notably occurred in a man. One key lesson is the utility of the PLASMIC score, especially for patients that “do not read the textbook.” It may be very tempting to dismiss a diagnosis of TTP in a patient with ITP or any other condition that can explain isolated thrombocytopenia. More generally, it can also be tempting to dismiss a diagnosis of TTP in a patient who is not in the “usual” demographic for TTP, as TTP is over-represented in females, black females, and most often afflicts such patients who are in their thirties and forties [24].

While the specificity of the PLASMIC score is moderate, the sensitivity is high. High sensitivity tests and scoring systems can be particularly useful when your goal is not to miss a high stakes diagnosis, especially when the therapy is relatively safe. Given TTP's stakes, urgency, and bespoke treatment, this case underscores the importance of treating empirically with TPE as soon as possible when the PLASMIC score is high—even when one or more patient characteristics or diagnoses tempt you to reconsider.

## Data Availability

No data sets were used other than the patient's medical record.

## References

[1] Zhang H. Y., Hou M., Zhang X. H., Guan X. H., Sun G. Z. (2004). The diagnostic value of platelet glycoprotein-specific autoantibody detection in idiopathic thrombocytopenic purpura. *Zhongguo Shi Yan Xue Ye Xue Za Zhi*.

[2] George J. N. (2010). How I treat patients with thrombotic thrombocytopenic purpura: 2010. *Blood*.

[3] Olsson B., Andersson P.-O., Jernås M. (2003). T-cell-mediated cytotoxicity toward platelets in chronic idiopathic thrombocytopenic purpura. *Nature Medicine*.

[4] Khodadi E., Asnafi A. A., Shahrabi S., Shahjahani M., Saki N. (2016). Bone marrow niche in immune thrombocytopenia: a focus on megakaryopoiesis. *Annals of Hematology*.

[5] Cines D. B., Bussel J. B., Liebman H. A., Luning Prak E. T. (2009). The ITP syndrome: pathogenic and clinical diversity. *Blood*.

[6] Amorosi E. L., Ultmann J. E. (1966). Thrombotic thrombocytopenic purpura. *Medicine*.

[7] Lesesve J.-F., Salignac S., Lecompte T. (2007). Laboratory measurement of schistocytes. *The International Journal of Literary Humanities*.

[8] Daram S. R., Philipneri M., Puri N., Bastani B. (2005). Thrombotic thrombocytopenic purpura without schistocytes on the peripheral blood smear. *Southern Medical Journal*.

[9] Tsai H. M., Raoufi M., Zhou W. (2006). ADAMTS13 and microvascular thrombosis. *Thrombosis and Haemostasis*.

[10] Scully M., Yarranton H., Liesner R. (2008). Regional UK TTP registry: correlation with laboratory ADAMTS 13 analysis and clinical features. *British Journal of Haematology*.

[11] Paydary K., Banwell E., Tong J., Chen Y., Cuker A. (2020). Diagnostic accuracy of the PLASMIC score in patients with suspected thrombotic thrombocytopenic purpura: a systematic review and meta‐analysis. *Transfusion*.

[12] Stein R. S., Flexner J. M. (1984). Idiopathic thrombocytopenic purpura during remission of thrombotic thrombocytopenic purpura. *Southern Medical Journal*.

[13] Baron B. W., Martin M. S., Sucharetza B. S., Jeon H.-R., Baron J. M. (2001). Four patients with both thrombotic thrombocytopenic purpura and autoimmune thrombocytopenic purpura: the concept of a mixed immune thrombocytopenia syndrome and indications for plasma exchange. *Journal of Clinical Apheresis*.

[14] Al-Husban N., Al-Kuran O. (2018). Post-partum thrombotic thrombocytopenic purpura (TTP) in a patient with known idiopathic (immune) thrombocytopenic purpura: a case report and review of the literature. *Journal of Medical Case Reports*.

[15] Takagi Y., Adachi Y., Tsujimura A., Tsushita K. (2012). Successful delivery following treatment with plasma exchange in a female patient with thrombotic thrombocytopenic purpura. *Rinsho Ketsueki*.

[16] Routy J.-P., Beaulieu R., Monte M., Saint-Louis J., Sauvageau G., Toma E. (1991). Immunologic thrombocytopenia followed by thrombotic thrombocytopenic purpura in two HIV1 patients. *American Journal of Hematology*.

[17] Prasad V. K., Kim I. K., Farrington K., Bussel J. B. (1996). TTP following ITP in an HIV-positive boy. *Journal of Pediatric Hematology*.

[18] Yospur L. S., Sun N. C. J., Figueroa P., Niihara Y. (1996). Concurrent thrombotic thrombocytopenic purpura and immune thrombocytopenic purpura in an HIV-positive patient: case report and review of the literature. *American Journal of Hematology*.

[19] Farhat M. H., Kuriakose P., Jawad M., Hanbali A. (2012). Sequential occurrence of thrombotic thrombocytopenic purpura, essential thrombocythemia, and idiopathic thrombocytopenic purpura in a 42-year-old African-American woman: a case report and review of the literature. *Journal of Medical Case Reports*.

[20] Dai F., Yang G., Rao P. (2020). Clinical characteristics of secondary immune thrombocytopenia associated with primary sjögren’s syndrome. *Frontiers of Medicine*.

[21] Carvalho J. F., Shoenfeld Y. (2020). Sjögren’s syndrome associated with thrombotic thrombocytopenic purpura: a case-based review. *Rheumatology and Therapy*.

[22] Kravitz M. S., Shoenfeld Y. (2005). Thrombocytopenic conditions-autoimmunity and hypercoagulability: commonalities and differences in ITP, TTP, HIT, and APS. *American Journal of Hematology*.

[23] Martı´n-Nares E., Herna´ndez-Molina G. (2019). Novel autoantibodies in Sjogren’s syndrome: a comprehensive review. *Autoimmunity Reviews*.

[24] Lee W., Perimbeti S., Vazquez Martinez M. (2013). Higher incidence of TTP in african Americans and females: an analysis of demographics, cost and length of stay in teaching and nonteaching hospitals for thrombotic thrombocytopenic purpura between 1999 and 2013.

